# Functional response properties of VIP-expressing inhibitory neurons in mouse visual and auditory cortex

**DOI:** 10.3389/fncir.2015.00022

**Published:** 2015-05-22

**Authors:** Lukas Mesik, Wen-pei Ma, Ling-yun Li, Leena A. Ibrahim, Z. J. Huang, Li I. Zhang, Huizhong W. Tao

**Affiliations:** ^1^Zilkha Neurogenetic Institute, University of Southern CaliforniaLos Angeles, CA, USA; ^2^Neuroscience Graduate Program, University of Southern CaliforniaLos Angeles, CA, USA; ^3^Cold Spring Harbor Laboratory, Cold Spring HarborNY, USA; ^4^Department of Physiology and Biophysics, University of Southern CaliforniaLos Angeles, CA, USA; ^5^Department of Cell and Neurobiology, University of Southern CaliforniaLos Angeles, CA, USA

**Keywords:** receptive field property, visual receptive field, tonal receptive field, orientation selectivity, direction selectivity, frequency tuning, intensity selectivity, interneuron

## Abstract

Despite accounting for about 20% of all the layer 2/3 inhibitory interneurons, the vasoactive intestinal polypeptide (VIP) expressing neurons remain the least thoroughly studied of the major inhibitory subtypes. In recent studies, VIP neurons have been shown to be activated by a variety of cortico-cortical and neuromodulatory inputs, but their basic sensory response properties remain poorly characterized. We set out to explore the functional properties of layer 2/3 VIP neurons in the primary visual (V1) and primary auditory cortex (A1), using two-photon imaging guided patch recordings. We found that in the V1, VIP neurons were generally broadly tuned, with their sensory response properties resembling those of parvalbumin (PV) expressing neurons. With the exception of response latency, they did not exhibit a significant difference from PV neurons across any of the properties tested, including overlap index, response modulation, orientation selectivity, and direction selectivity. In the A1, on the other hand, VIP neurons had a strong tendency to be intensity selective, which is a property associated with a subset of putative pyramidal cells and virtually absent in PV neurons. VIP neurons had a best intensity that was significantly lower than that of PV and putative pyramidal neurons. Finally, sensory evoked spike responses of VIP neurons were delayed relative to pyramidal and PV neurons in both the V1 and A1. Combined, these results demonstrate that the sensory response properties of VIP neurons do not fit a simple model of being either PV-like broadly tuned or pyramidal-like narrowly tuned. Instead, the selectivity pattern varies with sensory area and can even be, as in the case of low sound intensity responsiveness, distinct from both PV and pyramidal neurons.

## Introduction

Inhibitory interneurons are known to play important roles in shaping sensory cortical processing (Isaacson and Scanziani, 2011; Zhang et al., 2011). While their low density and high diversity (Kawaguchi and Kubota, 1997; Markram et al., 2004; Ascoli et al., 2008) continues to pose challenges, the use of molecular markers to divide them into nearly non-overlapping groups has greatly simplified systematic investigations of their precise unction (Gonchar and Burkhalter, 1997; Rudy et al., 2011). Three such inhibitory subtypes that have been most widely used are the parvalbumin (PV), somatostatin (SOM), and the vasoactive intestinal polypeptide (VIP) expressing neurons. Together, these three types of neuron cover the great majority of the inhibitory population (Rudy et al., 2011).

In the sensory cortex, PV neurons integrate inputs from a broad range of surrounding excitatory neurons (Hofer et al., 2011). This connectivity pattern gives rise to their broad tuning and the ability to reflect the overall level of surrounding neuronal activity (Kerlin et al., 2010; Ma et al., 2010; but see Runyan et al., 2010). As such, PV neurons are thought to play a normalization role (Carandini and Heeger, 2011), decreasing the activity of excitatory neurons if the overall level of excitation is too strong while preserving their tuning (Atallah et al., 2012; Wilson et al., 2012; Lee et al., 2014). On the other hand, SOM neurons, which most notably include Martinotti cells that target distal dendrites of pyramidal neurons, are more sharply tuned and their functional properties in both the visual and auditory cortex somewhat resemble those of pyramidal neurons (Ma et al., 2010; Li et al., 2014a). It has previously been suggested that they mediate subtractive rather than divisive inhibition (Wilson et al., 2012).

More recently, several studies have suggested that the function of SOM neurons might be better understood by looking at when they get inactivated rather than when they get activated (Gentet et al., 2012; Kvitsiani et al., 2013; Lee et al., 2013). This inactivation is likely mediated through another major subtype of interneurons, the VIP expressing interneurons, which were found to preferentially inhibit SOM neurons (Pfeffer et al., 2013; Pi et al., 2013). VIP neurons are a subgroup of the diverse ionotropic serotonin receptor 5HT3a (5HT3aR) expressing group of neurons (Lee et al., 2010). They often co-express other markers that have previously been used to delineate inhibitory subtypes, including calretinin, cholecystokinin, and others (Cauli et al., 2000; Lee et al., 2010; Miyoshi et al., 2010; Xu et al., 2010). It is perhaps due to the obvious neuromodulatory association (Porter et al., 1999; Férézou et al., 2002, 2007; Lee et al., 2010; Alitto and Dan, 2012; Arroyo et al., 2012) that the VIP neurons have been mostly studied in the behavioral rather than sensory-processing context. In particular, they have recently been shown to be activated by reinforcement signals (reward and punishment), distinct behavioral states as well as by long range cortico-cortical inputs (Pi et al., 2013; Lee et al., 2013; Fu et al., 2014; Zhang et al., 2014). In these cases, activation of VIP neurons inhibits SOM neurons, resulting in an increase of excitatory neuron responses. The exact purpose of this circuit is still debated, but an immediate effect could be a temporary shift of inhibition from dendrites toward soma while in longer term the disinhibition could serve a permissive role in enabling plasticity on the distal dendrites (Letzkus et al., 2011; Jiang et al., 2013; Pfeffer et al., 2013). Whichever is the case, it is important that we not only understand the modulatory inputs to VIP neurons but also the driving inputs from the local sensory network that likely determine which subpopulation of VIP neurons gets activated during relevant behavioral events. While such basic characterization is readily available for PV and SOM neurons, VIP neurons remain poorly examined. In the present study we set out to fill in this gap by directly targeting genetically labeled VIP neurons using the two-photon imaging guided loose-patch recording method (Liu et al., 2009). We found that in the visual cortex VIP neurons were broadly tuned, functionally similar to PV neurons except for the delayed responses. On the other hand, in the auditory cortex VIP neurons had the surprising tendency to be intensity selective, which clearly distinguishes them from auditory PV neurons.

## Materials and Methods

### Animal Preparation

All experimental procedures used in this study were approved under the Animal Care and Use Committee at the University of Southern California. The VIP-ires-Cre (Taniguchi et al., 2011) or PV-Cre driver line was crossed with the Ai14 (td-Tomato) reporter line (The Jackson laboratory). Animals were housed in a vivarium with a 12/12-h light/dark circle. Adult female mice (C57BL6 background, 2–3 months old) were sedated with chlorprothixene (0.05 mL of 4 mg/mL) and anesthetized with urethane (1.2 g/kg). Local anesthesia was applied by administrating bupivacaine subcutaneously. The body temperature was maintained at 37.50°C by a feedback heating system (Harvard Apparatus, Holliston, MA, USA). Surgical procedure was performed to expose the visual cortex or auditory cortex as previously described (Niell and Stryker, 2008; Liu et al., 2009, 2010; Li et al., 2014b). Eyelids were sutured during the surgical procedure. For recording visual responses, the right eyelid was re-opened after the surgery. The eye was rinsed with saline and a thin layer of silicone oil (30,000 centistokes) was applied to prevent drying while allowing clear optical transmission. Multiunit recordings were performed to determine the retinotopic map and location of the primary visual (V1) or primary auditory cortex (A1), as previously described (Liu et al., 2009, 2010; Li et al., 2014b). Two-photon guided recordings were performed immediately after the pre-mapping. Throughout the procedure, the depth of anesthesia was monitored by regular toe pinches, and if necessary, the mouse was supplemented with 20% of the original dose of urethane.

### *In Vivo* Two-Photon Imaging Guided Recording

*In vivo* two-photon imaging was performed with a custom-built imaging system. A mode-locked Ti:sapphire laser (MaiTai Broadband, Spectra-Physics) was tuned at 910 nm with the output power at 10–30 mW for layer 2/3 neurons at a depth from 150 to 300 μm. Scanning was controlled by a custom-modified scanning software (Scanimage 3.5, from Dr. K. Svoboda’s Laboratory, Janelia Farm, Ashburn, VA, USA; Pologruto et al., 2003). The depth of the patched cell was directly determined under imaging. For cell-attached recording, the glass electrode, with 8–10 MΩ impedance, was filled with a potassium-based intrapipette solution (in mM): 125 K-gluconate, 4 MgATP, 0.3 GTP, 10 phosphocreatine, 10 HEPES, 1 EGTA, pH 7.2, and 0.15 mM calcein (Invitrogen). The pipette tip was navigated in the cortex and patched onto a fluorescent soma as previously described (Liu et al., 2009). After confirming a successful targeting (Liu et al., 2009), a loose seal was formed (with 100–500 MΩ resistance) and maintained throughout the course of the recording. Spike responses were recorded with an Axopatch 200B amplifier (Molecular Devices). Loose-patch recording was made under voltage-clamp mode and the command potential was adjusted so that the baseline current was close to 0 pA. The recorded signal was filtered at 10 kHz and sampled at 20 kHz.

### Visual Stimulation

Software for visual stimulation was custom-developed using LabView (National Instruments) and MATLAB (MathWorks). Visual stimuli were provided by a 34.5 cm × 25.9 cm monitor (refresh rate 75 Hz, mean luminance ∼12 cd/m^2^) placed 25 cm away from the right eye. The center of the monitor was placed at 450° azimuth (corresponding to the monocular zone), 00° elevation, and it covered ±350° horizontally and ±270° vertically of the visual field of the mouse. To map spatial receptive fields (RFs), bright and dark squares over a gray background (contrast 70 and -70%, respectively) within an 11 × 11 grid (grid size 50°) were flashed individually (duration = 200 ms, interstimulus interval = 300 ms) in a pseudo-random sequence. The sign of contrast (On or Off) was determined randomly. Each location was stimulated for 8–24 times, and the same number of On and Off stimuli were applied. The On and Off subfields were derived from responses to the onset of bright and dark stimuli, respectively. To measure orientation tuning, drifting sinusoidal gratings of 12 directions (300° step) with temporal frequency of 2 Hz and spatial frequency 0.04 cycle/0° were presented on the full screen for 2 s with an interstimulus interval of 5.5 s. The grating started to drift 5 s after it appeared on the screen, and stopped drifting for 0.5 s. Grating of another orientation then appeared immediately. The mean luminance of the screen was thus kept constant. The 12 patterns were presented in a random sequence, and were repeated 5–10 times. For the measurement of response modulation, drifting sinusoidal gratings of preferred direction (with temporal frequency of 2 Hz) were presented for 50–100 cycles, at various spatial frequencies (0.01, 0.02, 0.04, 0.08, 0.16, 0.32 cycle/0°).

### Visual Data Analysis

For flash stimuli, stimulus-evoked spikes were counted within a 150 ms time window starting at the response onset. To quantify the evoked firing rate for each cell, responses to 4–5 flash stimuli at the RF center were selected to calculate an average firing rate (with baseline subtracted). For drifting gratings, spikes were counted within a 70–2000 ms window after the onset of the drift. The baseline activity (average spike number in the same length of duration before the onset of stimuli) was subtracted from stimulus-evoked spike numbers. To analyze RF structure, subfield was identified as an area where pixels with significant evoked responses (with peak firing rate larger than 3 SDs of baseline activity) were spatially contiguous. On and Off subfields were fitted with ellipses. The outline of the ellipse was determined as such that it could cross as many pixels at the boundary as possible. An overlap index (OI; Hirsch et al., 2003) was calculated for cells exhibiting both On and Off subfields. The OI is defined as:

$$OI=\frac{0.5{\;W}_{1}+0.5{\;\;W}_{2}-d}{0.5\;W_{1}+0.5\;W_{2}+d}$$

where *d* is the distance between the centers of two ellipses, *W*_1_ and*W*_2_ are the widths of them, respectively, which are the segments of the line that connects the two centers intercepted by the ellipses. The modulation ratio *M* = *R*(*F*_1_)/*R*(*F*_0_) was calculated for responses to gratings at optimal spatial frequency. The post-stimulus spike time histogram (PSTH) was first generated from all the cycles for responses over multiple repetitions. *R*(*F*_1_) was calculated from the PSTH as the amplitude of the best-fitting sinusoid at the modulation frequency (Mata and Ringach, 2005). *R*(*F*_0_) was the mean spike rate during the drifting grating stimulus (baseline subtracted). The strength of orientation selectivity was quantified with a global measure of orientation selectivity (Dragoi et al., 2000):

$$Global\,\;\;\;Orientation\,\;\;\;Selectivity\,\;\;\;index\,\;\;\;\;(OSI)=\sqrt{(\Sigma_{i}(R(\theta_{i})*\sin(2\theta_{i})){)}^{2}+(\Sigma_{i}(R(\theta_{i})*\sin(2\theta_{i})){)}^{2}}/\Sigma_{i}\,\;\;\;R(\theta_{i})$$

where, θ_i_ is the angle of the moving direction of the grating. *R*(θ_i_) is the spike response amplitude (with baseline subtracted) at angle θ_i_. The direction selectivity index (DSI) was defined as (*R*_pref_ -*R*_null_)/(*R*_pref_ + *R*_null_), *R*_pref_ is the maximum response and *R*_null_ is the response at the opposite direction. To quantify the onset latency of evoked responses, PSTH was generated from spikes evoked by all the flash stimuli (bin size = 4 ms). The onset of spiking responses was defined as the time point at which firing rate exceeded 3 SDs of baseline activity.

### Auditory Stimulation and Data Analysis

All the auditory experiments were carried out in a sound-attenuation room. The left auditory cortex was exposed, and the ear canal on the same side was plugged with a piece of clay wrapped with a thin layer of cotton. Tone pips (50-ms duration, 3-ms ramp) of various frequencies (2–32 kHz, 0.1 octave interval) and intensities [10–70 dB sound pressure level (SPL), at 10-dB interval] were generated by a custom software (LabView, National Instruments) through a 16-bit National Instruments interface, and delivered through a calibrated speaker (DT Tucker-Davis Technologies) to the contralateral ear. The 287 testing stimuli were presented in a pseudo-random sequence. All the experiments were carried out in the low-to-middle frequency regions (representing ∼5–20 kHz; identified during pre-mapping) of the A1. Tone-driven spikes were counted within a 10 to 110-ms time window after the onset of tones. Tonal receptive fields (TRFs) were reconstructed according to the array sequence. The spectral resolution of the frequency–intensity space was tripled by first dividing each bin into three sub-bins with the same value and next smoothing/weighted averaging with a (0.5, 1, 0.5) kernel. Boundaries of the spike TRF were determined following previous descriptions (Sutter and Schreiner, 1991; Schumacher et al., 2011). In short, a threshold at the value equal to the spontaneous spike rate plus 20% of the peak-evoked rate was used to define significant evoked responses. Responses to frequency-intensity combinations that met this criterion were considered to fall within the TRF of the neuron, which generated the contour of the TRF (Sun et al., 2010, 2013; Xiong et al., 2013; Li et al., 2014b). Intensity threshold was defined as the minimum intensity to evoke a significant excitatory response. Characteristic frequency (CF) was defined as the frequency (Hz) at which the lowest SPL was necessary to evoke a significant excitatory response. In the case of PV cells which exhibited broad frequency tuning even at the intensity threshold, the frequency at the center of the frequency responding range was chosen as the CF. Bandwidth (BW) of TRF was determined as the total frequency range for effective tones, at the intensity level of 10 dB above the threshold (i.e., BW10), with the exception of several intensity-selective VIP neurons which responded poorly at 10-dB above the threshold. For the latter neurons, we used BW directly at the threshold instead. To generate an intensity tuning curve, spike rates at the CF and two neighboring frequencies were averaged for each tone intensity. Intensity selectivity index (ISI) was calculated as 1 minus the ratio between the spike count at 30 dB above the best intensity (i.e., the intensity that produced maximum spike count) or the highest intensity tested and that at the best intensity. Onset latency of spike response was determined from the generated PSTH as the lag between the stimulus onset and the time point where spike rate exceeded the average baseline by 3 SDs of baseline fluctuations.

### Statistical Analysis

All of the statistical testing was done in matlab and involved comparisons between VIP, putative pyramidal, and PV neurons. We first used the Shapiro–Wilk test to check whether the distributions for the tested property are close to normal for all the groups. In the case they were, we tested for difference in means using ANOVA, otherwise we tested for difference in medians using the non-parametric Kruskal–Wallis test. Rejection of null hypothesis by ANOVA was followed by Tukey–Kramer multiple comparisons test to find significant differences between groups. Rejection of null hypothesis by Kruskal–Wallis was followed by Dunn’s multiple comparisons test. All of the reported *p*-values are after the multiple comparisons adjustments.

## Results

### General Characterization of VIP Neurons in the Primary Visual and Auditory Cortex

**FIGURE 1:**
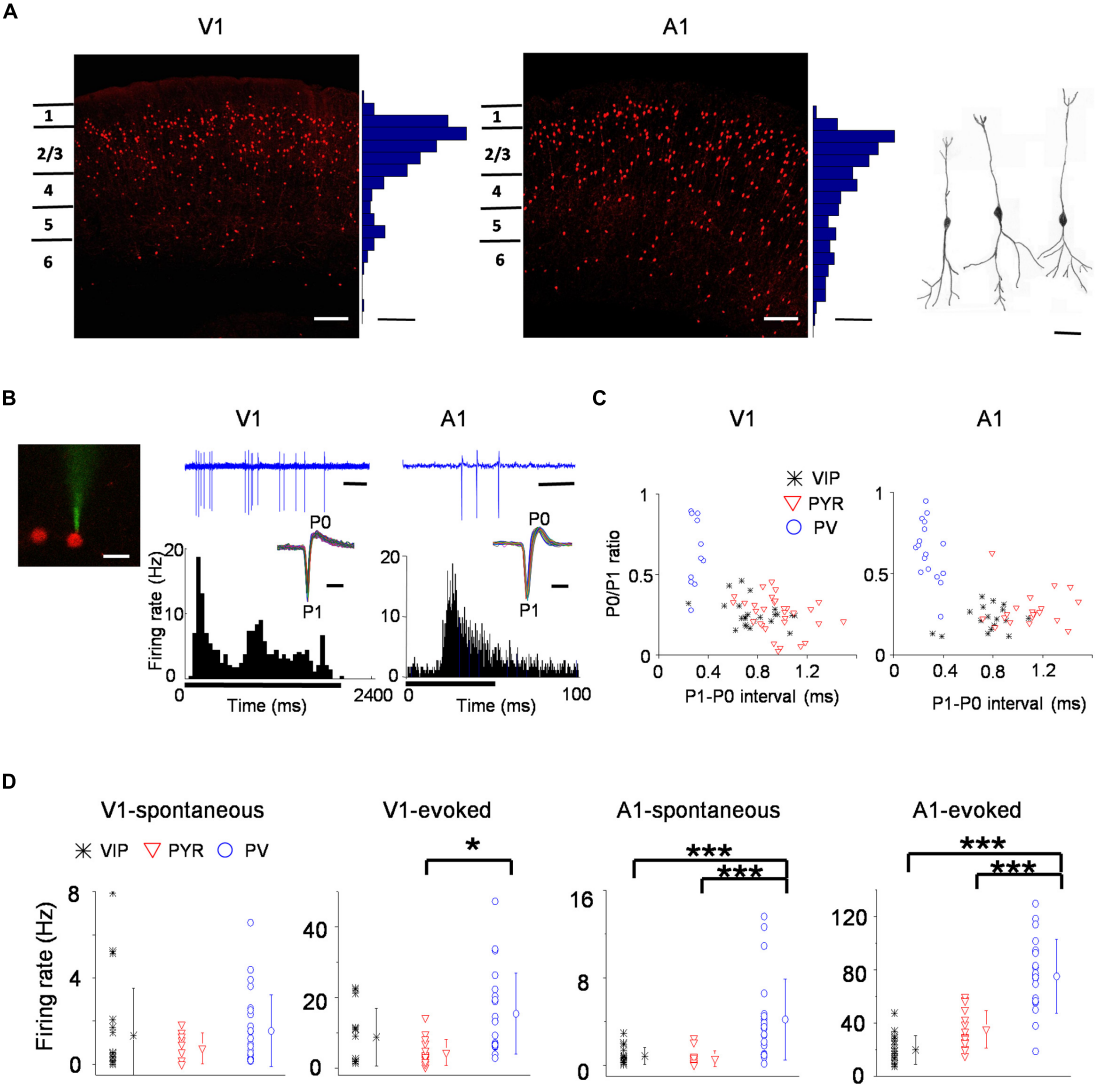
**Spiking properties of vasoactive intestinal polypeptide (VIP) neurons in the primary visual (V1) and primary auditory cortex (A1)**. **(A)** Coronal sections of a VIP-Cre::tdTomato brain showing red fluorescence labeled VIP neurons in the V1 (left) and A1 (right). Scale: 150 μm. Histograms adjacent to the images show the distribution of fluorescent cell bodies by depth within 50 μm bins. Scale: three cells per 50 μm × 00 μm × 100 μm. Far right, reconstructed morphologies of three representative VIP cells. Scale: 30 μm. **(B)** Left, a two-photon image showing a calcein-filled glass pipette forming a loose seal with a targeted VIP neuron. Scale: 20 μm. Middle, a sample recorded spike-response trace (2.4 s duration, scale: 300 ms; note that each vertical deflection reflects a spike current) of an example VIP neuron to a drifting grating (upper) and the post-stimulus spike time histogram (PSTH; lower) made for responses to all 12 orientations and of 5 repetitions. The black bar below indicates duration of the stimulus (onset at 0 s, 2 s long). Inset, superimposed individual spike waveforms during the recording for the displayed example cells. The trough and peak are labeled as P1 and P0, respectively. Scale: 1 ms. Right, tone-evoked responses of an example VIP neuron in the A1. The duration of the sample response trace is 100 ms (scale bar: 20 ms). The PSTH was made for responses to effective tones (38 tone–intensity combinations) and of nine repetitions. Black bar below indicates duration of the stimulus (onset at 0 s, 50 ms long). Scale for inset: 1 ms. **(C)** Scatterplots of P0/P1 amplitude ratio versus P1–P0 interval (a measure of spike width) for VIP (black), putative pyramidal (red) and PV (blue) cell populations. Each data point represents one cell. **(D)** Summaries of spontaneous and evoked firing rates of different cell populations. Mean values ± SD are shown for each cell group. Cell number: *n* = 20 in V1 and 17 in A1 for VIP; *n* = 22 in V1 and 22 in A1 for PV; 17 in V1 and 16 in A1 for Pyr. ^∗^*p* < 0.05; ^∗∗∗^*p* < 0.001, Kruskal–Wallis (except for A1-evoked responses where ANOVA was used) and *post hoc* test.

To study VIP neurons, we genetically labeled VIP-positive GABAergic neurons by crossing VIP-Cre driver mice (Taniguchi et al., 2011) with Ai14, a Cre-dependent TdTomato reporter line (Madisen et al., 2012). This resulted in labeling of VIP neurons with red fluorescence. Labeled VIP neurons are the densest in layer 2/3 of both the V1 and A1, although they are present in both upper and deep layers of the sensory cortices (**Figure 1A**, left and middle panel). Consistent with previous observations (Taniguchi et al., 2011), these VIP neurons exhibited a bipolar morphology (**Figure 1A**, right panel). Using a previously described method (Liu et al., 2009), we obtained *in vivo* cell-attached loose-patch recordings from fluorescence labeled neurons in layer 2/3 of either V1 or A1. After forming a loose seal on a targeted cell (**Figure 1B**, left panel), we presented visual (flash squares and drifting gratings) or auditory (pure tones) stimuli depending on the targeted cortical region, and recorded the cell’s spike responses to sensory stimulation (**Figure 1B**, middle and right panels). For each individual stimulus, responses of 5–15 repetitions were obtained. Under our experimental condition, the shape of recorded spikes remained reasonably stable throughout the recording session (**Figure 1B**, insets in the middle and right panels), allowing us to characterize basic spike shape properties such as trough-to-peak interval (i.e., P1–P0 interval) and peak/trough amplitude ratio (i.e., P0/P1 ratio). To compare VIP neurons to other cell types, we also recorded from fluorescence labeled PV expressing neurons in PV-Cre::Ai14 mice, and unlabeled neurons (putative excitatory/pyramidal neurons) in these mice.

As shown in **Figure 1C**, the PV and putative excitatory neuron populations were segregated according to P1–P0 interval. PV neurons showed shorter P1–P0 intervals than the putative excitatory cells, and they also tended to have higher P0/P1 ratios. The majority of VIP neurons had spike shapes resembling those of putative pyramidal cells (**Figure 1C**). Since there was a large overlap of P1–P0 interval between the VIP and putative excitatory neuron populations, spike waveforms of VIP neurons were in general individually indistinguishable from putative excitatory cell spikes, although on average they were significantly narrower compared to putative excitatory cells (P1–P0 interval: 0.76 ± 0.19 ms versus 1.0 ± 0.23 ms, *p* < 0.005, two-tailed *t*-test). Additionally, there were a few outliers which had narrow spikes similar to PV cells, but these VIP cells tended to have lower P0/P1 ratios than PV neurons (**Figure 1C**). Such trend was observed in both the V1 and A1.

We also compared the overall level of spiking activity of VIP neurons to PV and putative excitatory cells (**Figure 1D**). In the V1, the spontaneous spike rate of VIP neurons was comparable to that of PV cells, whereas in the A1 it was significantly lower than that of PV cells (V1: 1.3 ± 2.2 Hz for VIP, 1.5 ± 1.7 Hz for PV; A1: 0.85 ± 0.78 Hz for VIP, 4.2 ± 3.7 Hz for PV). The spontaneous rate of putative pyramidal cells was in general lower (0.74 ± 0.71 Hz in V1; 0.59 ± 0.72 Hz in A1). The evoked spike rate of VIP neurons was not significantly different from that of putative pyramidal neurons in either V1 or A1 (V1: 8.7 ± 8.2 Hz for VIP, 4.4 ± 3.7 Hz for Pyr; A1: 19.7 ± 10.8 Hz for VIP, 35.3 ± 14 Hz for Pyr). On the other hand, the evoked activity of PV neurons was higher than both cell types in the V1 and A1 (V1:15.4 ± 11.5 Hz; A1: 75 ± 28 Hz). Since SOM neurons inhibit VIP neurons (Pfeffer et al., 2013), the difference in the relative activity levels of VIP neurons in the V1 versus A1 could possibly be due to SOM neurons being generally more active in the auditory cortex than in the visual cortex of anesthetized animals (see Ma et al., 2010; Adesnik et al., 2012; Li et al., 2014a).

### Visual Response Properties of VIP Neurons

**FIGURE 2:**
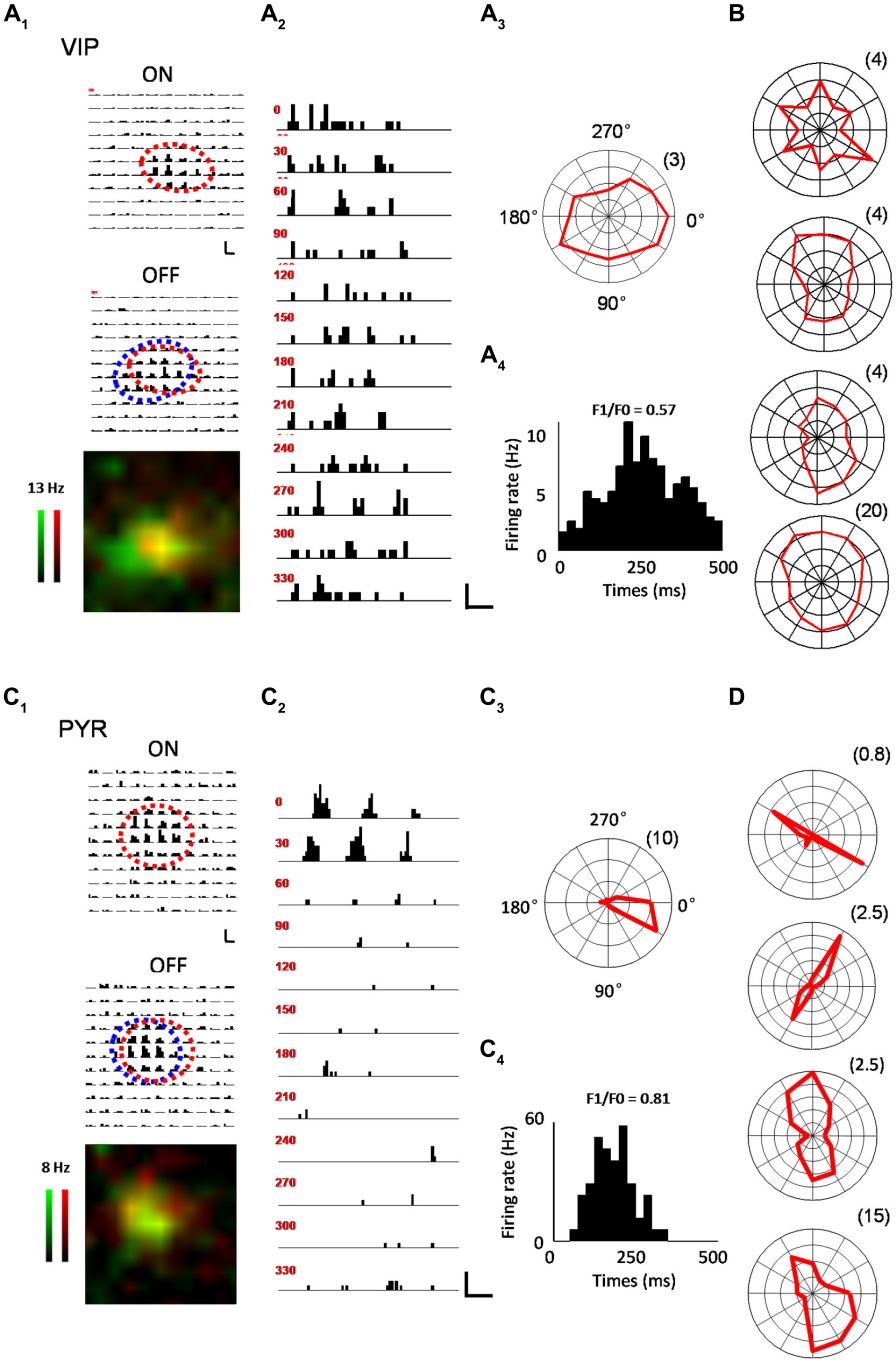
**Characterization of visual response properties. (A)** An example VIP neuron. **A_**1**_**, array of PSTHs for the responses to 11 × 11 flash bright (On, upper) and dark (Off, lower) squares of 10 repetitions. The duration of flashing stimuli is indicated by a red bar. Scale: 12 Hz, 200 ms. Fitted On and Off response regions are marked by red and blue ovals, respectively. Bottom, superimposed color maps for On (red) and Off (green) responses. Color scale represents evoked firing rate. **A_**2**_**, PSTHs for responses to drifting sinusoidal gratings at 12 different directions. Scale: 10 Hz, 400 ms. **A_**3**_**, Polar graph plot of orientation-dependent response levels. The radius of the polar plot (firing rate in Hz) is indicated within parentheses. **A_**4**_**, Cycle averaged PSTH for spike responses evoked by drifting sinusoidal grating at optimal direction and spatial frequency. The modulation ratio F1/F0 is indicated. **(B)** Polar graph plots for four more example VIP neurons. **(C)** An example pyramidal neuron. Data are displayed in the same manner as in **(A)**. Scale in **C_**2**_**: 33 Hz, 400 ms. **(D)** Polar graph plots for four more example pyramidal neurons.

In the V1, we were interested in several key receptive field properties that have previously been used to characterize functional responses of excitatory and inhibitory neurons (Liu et al., 2009; Ma et al., 2010). For a subset of recorded VIP neurons, we first applied a set of bright and dark flash squares to map the On and Off subfields, respectively (see Materials and Methods). As shown by an example VIP neuron in **Figure 2A**, the cell exhibited largely overlapped On and Off response regions (**Figure 2A_1_**), suggesting that it was a complex cell (Skottun et al., 1991; Niell and Stryker, 2008). We next measured its orientation tuning by presenting drifting sinusoidal gratings at 12 different directions (**Figure 2A_2_**). As shown by the polar graph plotting (**Figure 2A_3_**), the VIP neuron had a more or less clear orientation bias, but the level of tuning selectivity was rather weak. From the cycle-averaged PSTH for the responses at the optimal orientation/direction and spatial frequency, we measured the degree of response modulation by calculating the ratio of the modulation depth of the response (F1 component) to the elevation of the response (F0 component), i.e., the F1/F0 ratio (Skottun et al., 1991; see Materials and Methods). The VIP neuron’s response was only weakly modulated, as evidenced by the F1/F0 ratio smaller than 1 (**Figure 2A_4_**), which is consistent with the notion that the cell was a complex cell (Skottun et al., 1991; Niell and Stryker, 2008). Polar graphs for another four example VIP neurons are shown in **Figure 2B**. All these cells exhibited only weak orientation tuning. As a comparison, the On and Off subfields and orientation tuning of example putative pyramidal cells are shown in a similar manner in **Figures 2C,D**. Notably, the putative pyramidal cells looked much more sharply tuned for orientation than the VIP cells (**Figures 2C_3_,D**).

**FIGURE 3:**
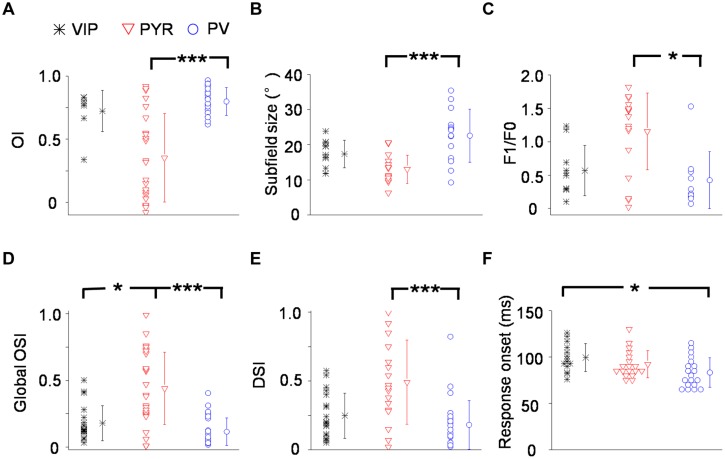
**Summary of visual response properties for VIP neurons as compared to PV and pyramidal neurons. (A)** Distribution of overlap indices (OI). Mean values ± SD are shown for cell groups. *N* = 8 for VIP; 23 for Pyr; 25 for PV. **(B)** Distribution of subfield sizes. **(C)** Distribution of modulation (F1/F0) ratios. *N* = 10 for VIP; 18 for Pyr; 10 for PV. **(D)** Distribution of global orientation selectivity indices (gOSI). **(E)** Distribution of direction selectivity indices (DSI). **(F)** Distribution of spiking response onset latencies. *N* = 14 for VIP; 18 for Pyr; 19 for PV. ^∗^*p* < 0.05; ^∗∗∗^*p* < 0.001, Kruskal–Wallis (except for **B**, where ANOVA was used) and *post hoc* test.

Out of 14 VIP neurons tested with flash stimuli 8 exhibited both ON and OFF spike subfields. The rest of the neurons (6/14) only displayed an OFF subfield. We fit each spike subfield with an ellipse. An OI was calculated to measure the spatial overlap between the On and Off subfields of an individual neuron (see Materials and Methods). As shown in **Figure 3A**, OIs of putative pyramidal cells spanned 0 and 1, consistent with the notion that these cells exhibited both simple- and complex-cell properties (Niell and Stryker, 2008; Liu et al., 2009). VIP neurons, however, exhibited mostly large OIs similar as PV cells, indicating a high degree of overlap between the On and Off response regions. Therefore VIP and PV neurons were mostly complex cells. VIP neurons tended to have slightly larger subfields than pyramidal cells (17.0 ± 4.00° versus 13.0 ± 4.00°, *p* = 0.09), with the latter having significantly smaller subfields compared to PV neurons (13.0 ± 4.00° versus 23.6 ± 7.50°, *p* < 0.001; **Figure 3B**). As for response modulation under sinusoidal gratings, VIP and PV neurons exhibited similar F1/F0 ratios, which tended to be lower than those of the putative pyramidal cells (**Figure 3C**), consistent with the finding that VIP and PV neurons mostly exhibited complex-cell properties (**Figure 3A**). Both VIP and PV neurons also had a lower OSI (**Figure 3D**) and a lower DSI (**Figure 3E**) as compared to the putative pyramidal cells, although in the case of DSI the difference between VIP and putative pyramidal cells did not reach significance (*p* = 0.063). All these data demonstrate that in the V1, the functional properties of VIP neurons, while appearing intermediate between pyramidal and PV neurons, more closely resemble those of PV cells. These two types of neuron both have weaker feature selectivity, including spatial, orientation, and direction selectivity, than pyramidal neurons.

Finally, we compared the onset timing of evoked responses of different cell types (**Figure 3F**). From the PSTH generated for responses to flash stimuli around the center of the subfield of the cell’s optimal contrast (On or Off), we determined the onset of evoked responses as the time point at which the firing rate exceeded the baseline by three SDs (see Materials and Methods). In general, there were large variations of response latency. Nonetheless, we found that the onset latency of VIP neuron responses tended to be longer than putative pyramidal cells and was significantly longer than PV neurons (99 ± 15 ms for VIP; 90 ± 15 ms for Pyr; 83 ± 16 ms for PV). Therefore, the evoked responses of VIP neurons are relatively delayed compared to the other two cell types.

### Auditory Response Properties of VIP Neurons

**FIGURE 4:**
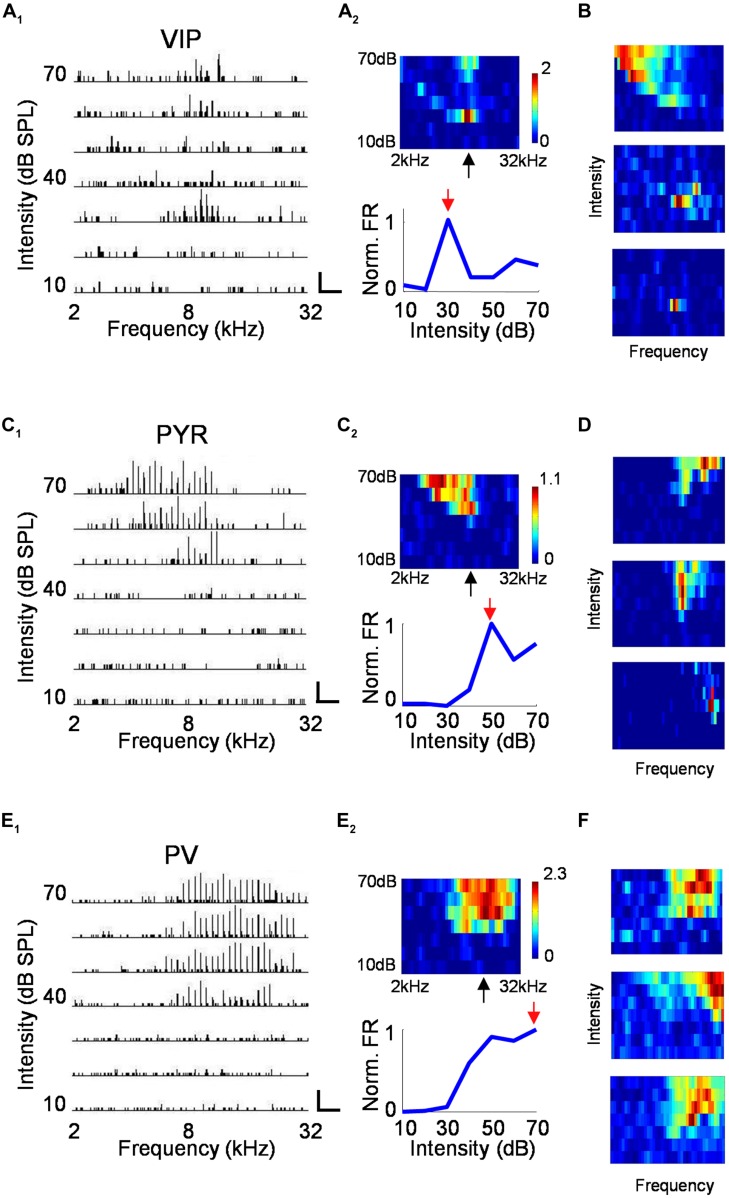
**Characterization of auditory response properties. (A)** An example VIP neuron. **A_****1****_**, tonal receptive field plotted as an array of PSTHs (five repetitions) to tones of varying intensity (10–70 dB SPL, 10 dB steps) and frequency (2–32 kHz, 0.1-octave steps). Scale: 29 Hz, 500 ms. Tone duration was 100 ms. **A_****2****_**, top, color map representation of the tonal receptive field (TRF). Pixel color represents the average number of spikes evoked per stimulus repetition within a 100-ms analysis window. Black arrow points to the characteristic frequency (CF). Bottom, response levels along the intensity, measured at and close to the CF and normalized by the maximum response level. Red arrow points to the best intensity. **(B)** Color maps for TRFs of another three example VIP neurons. Color scale (from top to bottom): 3.4; 1.3; 0.9 for maximum. **(C,D)** Example pyramidal neurons. Data are presented in the same manner as in **(A,B)**. Scale in **C_**1**_**: 50 Hz, 250 ms. Color scale for (**D**; from top to bottom): 2.8; 2.2; 1.8 for maximum. **(E,F)** Example PV neurons. Scale in **E_**1**_**: 75 Hz, 250 ms. Color scale for (**D**; from top to bottom): 4.2; 3.5; 2.8 for maximum.

For neurons recorded in the A1, we mapped the frequency–intensity TRF by presenting to the mouse tone pips of different frequencies (2–32 kHz) and intensities (10–70 dB SPL at 10 dB step). **Figure 4A_1_** plots the PSTHs for spike responses of a VIP neuron to all testing tone stimuli, arranged according to the corresponding frequency and intensity. The measured firing rates are displayed with a color map representing the frequency–intensity space (**Figure 4A_2_**, upper panel). Surprisingly, we found that many VIP neurons were strongly tuned for intensity, as evidenced by the greatly increased firing rate near the intensity threshold, which was then followed by lowered firing rates at higher sound intensities (**Figure 4A_2_**, lower panel). Additional example VIP TRFs are shown in **Figure 4B**. In extreme cases, intensity-tuned VIP neuron displayed an enclosed, “o”-shaped TRF (**Figure 4B**, middle and lower panel). While intensity-tuned TRFs were often observed for VIP neurons, they were not or rarely observed for putative pyramidal cells and PV neurons, which usually exhibited conventional “V” or “U”-shaped TRFs (**Figures 4C–F**). VIP neurons not exhibiting “o”-shaped TRFs tended to show broadly tuned TRFs which may resemble those of PV cells (**Figure 4B**, upper panel).

**FIGURE 5:**
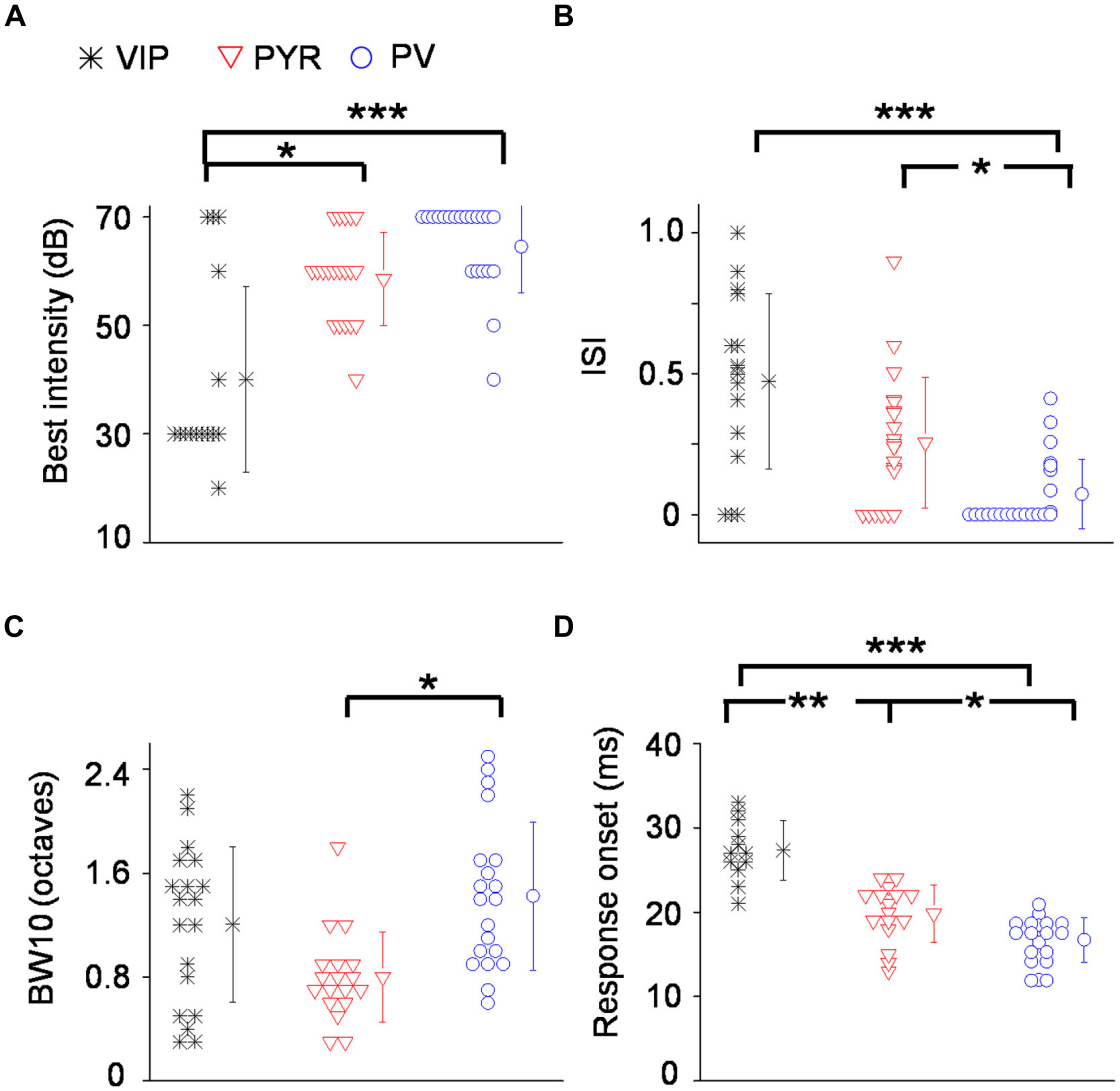
**Summary of auditory response properties of VIP neurons as compared to PV and pyramidal neurons. (A)** Distribution of best intensities. *N* = 16 for VIP; 21 for Pyr; 22 for PV. **(B)** Distribution of intensity selectivity indices (ISI). **(C)** Distribution of TRF bandwidths (BWs) measured at 10 dB above intensity threshold (or at the threshold). **(D)** Distribution of spiking response onset latencies. ^∗^*p* < 0.05; ^∗∗^*p* < 0.01; ^∗∗∗^*p* < 0.001, Kruskal–Wallis and *post hoc* test.

To quantify the degree of intensity tuning, we derived a rate-level function, which is defined as the mean evoked firing rate along all the testing intensities, confined to frequencies at and around the CF (see Materials and Methods). We used this function for two different measures. First, we determined the best intensity of the neuron, defined as the intensity at which the strongest response was observed. More than half of the VIP neurons responded most strongly at 30 dB SPL which was at or 10 dB above their absolute intensity threshold (**Figure 5A**). In comparison, the best intensity of putative pyramidal cells tended to be much higher at 50 or 60 dB SPL. The best intensity alone does not tell us how strongly the responses are modulated by intensity; for instance, the responses could peak at a low sound intensity and then stay the same at higher intensities. To assess the strength of intensity tuning, we calculated an intensity-selectivity index (ISI), defined as 1 minus (response at 30 dB above the best intensity or the highest intensity tested)/(response at the best intensity). For half of the VIP neurons (8 out of 16 cells, 50%), this index was above 0.5, meaning that these cells were intensity selective (**Figure 5B**). In comparison, the ISIs of putative pyramidal cells largely spread below 0.5, and the percentage of intensity-selective cells (3 out of 21 cells, 14.3%) was much lower compared to the VIP population. However, the ISI measure did not reveal a significant difference between VIP and putative pyramidal neurons (0.47 ± 0.3 for VIP; 0.26 ± 0.23 for Pyr, *p* = 0.13). One could therefore conclude that VIP neurons tend to reach peak responses at lower intensities than pyramidal neurons, but the strength of intensity selectivity is then similar between the two cell types. Among the three types of cell, PV neurons exhibited the weakest intensity tuning (ISI = 0.07 ± 0.1). None of the 22 recorded PV cells was intensity selective (with intensity-selectivity index >0.5).

Next, we measured the TRF BW. At 10 dB above the intensity threshold (or at the threshold, see Materials and Methods), VIP neurons tended to respond to a broader range of frequencies than pyramidal neurons (1.2 ± 0.6 octave versus 0.8 ± 0.35 octave, *p* = 0.06; **Figure 5C**). PV neurons displayed significantly broader BWs than pyramidal cells (1.4 ± 0.6 octave versus 0.8 ± 0.35 octave, *p* < 0.05; **Figure 5C**). Thus, similar to PV cells, VIP neurons tended to be broadly tuned, although some of their TRFs were enclosed.

Finally, we characterized the onset latency of spike responses to tones (**Figure 5D**). VIP neurons responded significantly later than putative pyramidal neurons (27.3 ± 3.6 ms versus 19.8 ± 3.4 ms, *p* < 0.001). Consistent with previous observations (Wu et al., 2008; Moore and Wehr, 2013), PV neurons responded significantly earlier than putative pyramidal cells (16.7 ± 2.6 ms versus 19.8 ± 3.4 ms, *p* < 0.05). On average, VIP neurons responded 7.5 ms later than putative pyramidal neurons, 10.6 ms later than PV neurons and within a single millisecond of SOM neuron firing (see Li et al., 2014a). Therefore, the responses of VIP neurons in the A1 are much delayed relative to pyramidal and PV cells, and instead appear to be aligned with SOM neuron responses.

## Discussion

**FIGURE 6:**
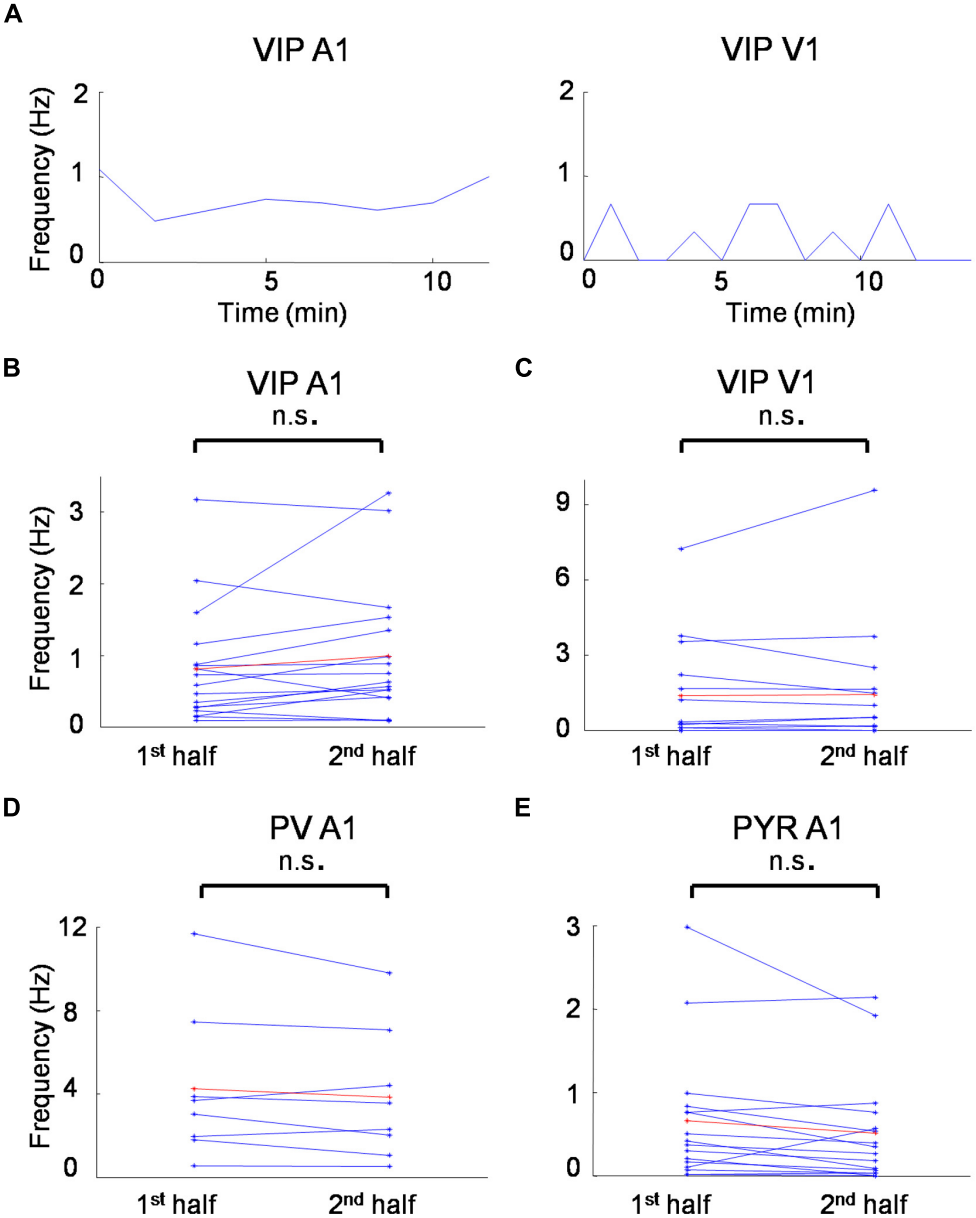
**Spontaneous firing rates during recording sessions. (A)** Time course of average spontaneous firing rates for a VIP neuron in the A1 (left) and a VIP neuron in the V1 (right). Each data point is the measure of average spontaneous firing rate during the receptive field mapping of one repetition. **(B)** Average firing rates for the first and second half duration of the recording session for all the VIP cells recorded in the A1. **(C)** Average firing rates for the first and second half duration of the recording session for all the VIP cells recorded in the V1. **(D)** Average firing rates for the first and second half duration of the recording session for all the PV cells recorded in the A1. **(D)** Average firing rates for the first and second half duration of the recording session for all the putative pyramidal cells recorded in the A1. n.s., non-significant, paired *t*-test.

Understanding receptive field properties of different inhibitory cell types is crucial for unraveling their specific roles in sensory processing. In this study we characterized the sensory receptive field properties of VIP-expressing inhibitory neurons in the mouse visual and auditory cortex and compared them to those of putative pyramidal neurons and PV cells, with the latter being the most prominent inhibitory cell type in the cortex. The average spontaneous firing rate was not significantly changed from the first to second half duration of recording for all the cell groups we recorded (**Figure 6**), indicating that the recording quality was relatively stable in our experimental conditions. We found that VIP neurons are robustly driven by sensory inputs, and that they are in general more broadly tuned than pyramidal neurons, similar as PV cells. However, VIP neurons also exhibit functional selectivity distinct from PV cells.

### Broadly Tuned Properties in the Visual Cortex

In a previous study, orientation tuning properties of a small number of VIP neurons have been examined with Ca^2+^ imaging, and the cell type of these neurons was identified *post hoc* with immunostaining for VIP (Kerlin et al., 2010). One concern over Ca^2+^ imaging is the potential non-linearity which can distort measurements of tuning of neuronal populations (Nauhaus et al., 2012). Here, we applied two-photon imaging guided loose-patch recordings from genetically labeled VIP neurons, which allowed us to collect data from a much larger number of specific cells and to measure their spike responses more accurately. We demonstrate that VIP neurons functionally resemble PV cells. They both have overlapping On and Off subfields and weakly modulated responses to gratings, and are more weakly selective for orientation and direction than pyramidal cells. The observation that VIP and PV neurons are both broadly tuned is in fact in agreement with the previously reported Ca^2+^ imaging results (Kerlin et al., 2010).

It has been found that the difference between orientation selectivity levels of PV and pyramidal neurons in layer 2/3 of the V1 can be attributed at least partially to differential specificity of intralaminar excitatory connectivity: while pyramidal cells preferentially receive excitatory inputs with similar orientation preferences, such selectivity is absent for excitatory inputs to PV neurons (Hofer et al., 2011). It is likely that VIP neurons also pool local excitatory inputs somewhat indiscriminately, resulting in their broad tuning. The functional significance of such broad pooling could be for VIP neurons to detect the general activity level of local excitatory networks, and thus to regulate the local microvessel output by releasing VIP which is a vasoactive substance (Cauli et al., 2004).

It has been proposed that broadly tuned PV neurons provide excitatory cells with broad inhibitory inputs, which helps to sharpen the tuning of excitatory neurons (Ma et al., 2010; Liu et al., 2011; Atallah et al., 2012). We have also reported previously that SOM neurons, another major inhibitory cell type, are as sharply tuned for orientation as excitatory neurons (Ma et al., 2010). Since VIP neurons preferentially inhibit SOM neurons but rarely inhibit pyramidal cells (Pfeffer et al., 2013; Pi et al., 2013), it is likely that the broadly tuned VIP neurons provide broad inhibitory inputs to SOM neurons, allowing their orientation tuning to be sharpened. VIP and PV neurons may thus compose two inhibitory control systems to modulate tuning properties of SOM and excitatory neurons, respectively.

### Intensity Tuned Properties in the Auditory Cortex

In the auditory cortex, we found in surprise that many of the VIP neurons are intensity selective, whereas PV and pyramidal neurons are not or only occasionally selective. This distinct feature of VIP neurons raises a question of how enclosed tonal receptive fields can be constructed in this cell group. For excitatory neurons, previous studies in rodents suggest that intensity selectivity can be created or sharpened through specific excitatory and inhibitory interplay in which excitation and inhibition have differential intensity tuning functions and/or have intensity-dependent variations of their relative onset timings (Wu et al., 2006; Tan et al., 2007; Zhou et al., 2012). Since VIP neurons preferentially receive inhibition from SOM neurons (Pfeffer et al., 2013), it is possible that the SOM neuron inhibition helps to sculpt the enclosed TRFs out of otherwise rather broadly tuned TRFs of VIP neurons. From previous studies, we know that SOM neurons have a higher intensity threshold than PV and pyramidal neurons, and that they are predominantly monotonic (i.e., non-intensity-tuned) cells similar as PV cells (Li et al., 2014a). These properties indicate that SOM neurons are in a suitable position to provide the appropriate type of inhibition (i.e., with a higher intensity threshold compared to excitation, and monotonic intensity tuning) to facilitate the creation of intensity selectivity in VIP neurons. If SOM neurons are indeed important for generating intensity selectivity of VIP neurons, we would expect that this distinct property of VIP neurons can be disrupted when SOM neuron activity is reduced. Future investigations are needed to test this idea.

A second question is how the intensity selectivity of VIP neurons might be useful. The major function of layer 2/3 VIP neurons is to disinhibit pyramidal cells (Pfeffer et al., 2013; Pi et al., 2013). Since many VIP neurons respond strongly to low-intensity stimuli, an intriguing possibility is that these VIP neurons can help to resolve weak auditory stimuli by disinhibiting distal dendrites of pyramidal neurons. In fact, this could also serve as a mechanism for helping generate intensity selectivity in a subset of pyramidal cells. In addition, VIP neurons can be activated by a variety of cortico-cortical inputs and reinforcement signals (Lee et al., 2013; Pi et al., 2013; Fu et al., 2014; Zhang et al., 2014). Through these modulatory pathways, the identity of a barely detectable stimulus may be better resolved in a context or behavioral context dependent manner.

### Diversity within the VIP Population

While in the present study we treat VIP neurons as a single group, it is known that VIP as a marker labels neurons with a range of morphologies, intrinsic excitability properties and molecular co-expression patterns (Markram et al., 2004; Lee et al., 2010). Although the small number of VIP neurons we examined in detail showed a bipolar morphology, it is possible that our data also includes small basket cells (also previously described as ‘arcade cells’) that often co-express CCK (Kawaguchi and Kubota, 1996, 1997; Wang et al., 2002). A small subset of VIP neurons have narrow spike waveforms similar to PV cells, implying that these might be basket cells. However, we did not find these cells outliers in the distributions of functional properties. For example, one fast-spiking VIP neuron was intensity selective, while another was not intensity tuned. Moreover, even the typical bipolar VIP neurons may not be a homogeneous group (von Engelhardt et al., 2007). Despite this diversity within the VIP population, none of the properties we examined showed a strikingly double-peaked distribution that would imply two populations. Although it is possible that that this is at least partly due to small sample sizes, it nevertheless suggests that the majority of layer 2/3 VIP neurons within a given region share similar basic functional properties.

### Delayed Responses

In both the visual and auditory cortex, we found that the spiking responses of VIP neurons evoked by transient stimuli (flash and tone pips) tend to have longer onset delays compared to PV and pyramidal cells. There are several possible interpretations of this finding. First, our recent study of slice recording coupled with optogenetic stimulation indicates that layer 2/3 PV and pyramidal cells receive direct thalamic inputs in both the V1 and A1, whereas layer 2/3 VIP neurons do not receive direct thalamic inputs (Ji et al., 2015). The thalamic input may drive the earlier spiking of PV and pyramidal cells. Second, while the laminar pattern of local excitatory inputs to layer 2/3 bipolar (presumptive VIP) cells are similar to pyramidal neurons, the pattern of inhibitory inputs is different: VIP neurons appear to receive relatively stronger inhibition from layer 4, likely from PV neurons (Xu and Callaway, 2009). This inhibition from layer 4 may effectively prevent early spiking of VIP neurons. A third possibility is that the typical excitatory inputs VIP neurons receive from local sources may be generally weak, and cortico-cortical inputs from higher cortical areas or later responding subcortical areas tend to be more important drivers. While we lack precise information on the relative strength of local versus long range inputs to VIP neurons, there are known cases where VIP neurons receive stronger long range cortico-cortical inputs than pyramidal neurons (Lee et al., 2013; Zhang et al., 2014). A fourth possibility is that differential sensitivity of different cell types to anesthetics may contribute to their different response timings.

Finally, it should be noted that our experiments were performed in anesthetized mice, in which the cortical activity could be different from the way it normally functions in awake animals (Constantinople and Bruno, 2011). For example, in anesthetized mice SOM neurons are much less active than in some awake conditions (Adesnik et al., 2012). It will be necessary to systematically revisit these and other sensory response results in awake preparations and under specific behavioral contexts, considering the possible involvement of VIP neurons in the modulation of cortical responses by neuromodulatory and long range cortico-cortical inputs.

## Conflict of Interest Statement

The authors declare that the research was conducted in the absence of any commercial or financial relationships that could be construed as a potential conflict of interest.

## References

[B1] Adesnik H. Bruns W. Taniguchi H. Huang Z. J. Scanziani M. (2012). A neural circuit for spatial summation in visual cortex. *Nature* 490 226–231. 10.1038/nature11526 23060193 PMC3621107

[B2] Alitto H. J. Dan Y. (2012). Cell-type-specific modulation of neocortical activity by basal forebrain input. *Front. Sys. Neurosci.* 6:79 10.3389/fnsys.2012.00079 PMC3540901 23316142

[B3] Arroyo S. Bennett C. Aziz D. Brown S. P. Hestrin S. (2012). Prolonged disynaptic inhibition in the cortex mediated by slow, non-α7 nicotinic excitation of a specific subset of cortical interneurons. *J. Neurosci.* 32 3859–3864. 10.1523/JNEUROSCI.0115-12.2012 22423106 PMC3320796

[B4] Ascoli G. A. Alonso-Nanclares L. Anderson S. A. Barrionuevo G. Benavides-Piccione R. Burkhalter A. (2008). Petilla terminology: nomenclature of features of GABAergic interneurons of the cerebral cortex. *Nat. Rev. Neurosci.* 9 557–568. 10.1038/nrn2402 18568015 PMC2868386

[B5] Atallah B. V. Bruns W. Carandini M. Scanziani M. (2012). Parvalbumin-expressing interneurons linearly transform cortical responses to visual stimuli. *Neuron* 73 159–170. 10.1016/j.neuron.2011.12.013 22243754 PMC3743079

[B6] Carandini M. Heeger D. J. (2011). Normalization as a canonical neural computation. *Nat. Rev. Neurosci.* 13 51–62. 10.1038/nrn3136 22108672 PMC3273486

[B7] Cauli B. Porter J. T. Tsuzuki K. Lambolez B. Rossier J. Quenet B. (2000). Classification of fusiform neocortical interneurons based on unsupervised clustering. *Proc. Natl. Acad. Sci. U.S.A.* 97 6144–6149. 10.1073/pnas.97.11.6144 10823957 PMC18572

[B8] Cauli B. Tong X. K. Rancillac A. Serluca N. Lambolez B. Rossier J. (2004). Cortical GABA interneurons in neurovascular coupling: relays for subcortical vasoactive pathways. *J. Neurosci.* 24 8940–8949. 10.1523/JNEUROSCI.3065-04.2004 15483113 PMC6730057

[B9] Constantinople C. M. Bruno R. M. (2011). Effects and mechanisms of wakefulness on local cortical networks. *Neuron* 69 1061–1068. 10.1016/j.neuron.2011.02.040 21435553 PMC3069934

[B10] Dragoi V. Sharma J. Sur M. (2000). Adaptation-induced plasticity of orientation tuning in adult visual cortex. *Neuron* 28 287–298. 10.1016/S0896-6273(00)00103-3 11087001

[B11] Férézou I. Cauli B. Hill E. L. Rossier J. Hamel E. Lambolez B. (2002). 5-HT3 receptors mediate serotonergic fast synaptic excitation of neocortical vasoactive intestinal peptide/cholecystokinin interneurons. *J. Neurosci.* 22 7389–7397. 12196560 10.1523/JNEUROSCI.22-17-07389.2002 PMC6757992

[B12] Férézou I. Hill E. L. Cauli B. Gibelin N. Kaneko T. Rossier J. (2007). Extensive overlap of mu-opioid and nicotinic sensitivity in cortical interneurons. *Cereb. Cortex* 17 1948–1957. 10.1093/cercor/bhl104 17068095

[B13] Fu Y. Tucciarone J. M. Espinosa J. S. Sheng N. Darcy D. P. Nicoll R. A. (2014). A cortical circuit for gain control by behavioral state. *Cell* 156 1139–1152. 10.1016/j.cell.2014.01.050 24630718 PMC4041382

[B14] Gentet L. J. Kremer Y. Taniguchi H. Huang Z. J. Staiger J. F. Petersen C. C. (2012). Unique functional properties of somatostatin-expressing GABAergic neurons in mouse barrel cortex. *Nat. Neurosci.* 15 607–612. 10.1038/nn.3051 22366760

[B15] Gonchar Y. Burkhalter A. (1997). Three distinct families of GABAergic neurons in rat visual cortex. C*ereb. Cortex* 74 347–358. 10.1093/cercor/7.4.347 9177765

[B16] Hirsch J. A. Martinez L. M. Pillai C. Alonso J. M. Wang Q. Sommer F. T. (2003). Functionally distinct inhibitory neurons at the first stage of visual cortical processing. *Nat. Neurosci.* 6 1300–1308. 10.1038/nn1152 14625553

[B17] Hofer S. B. Ko H. Pichler B. Vogelstein J. Ros H. Zeng H. (2011). Differential connectivity and response dynamics of excitatory and inhibitory neurons in visual cortex. *Nat. Neurosci.* 14 1045–1052. 10.1038/nn.2876 21765421 PMC6370002

[B18] Isaacson J. S. Scanziani M. (2011). How inhibition shapes cortical activity. *Neuron* 72 231–243. 10.1016/j.neuron.2011.09.027 22017986 PMC3236361

[B19] Ji X. Y. Zingg B. Mesik L. Xiao Z. Zhang L. I. Tao H. W. (2015). Thalamocortical innervation pattern in mouse auditory and visual cortex: laminar and cell-type specificity. *Cereb. Cortex*. 10.1093/cercor/bhv099 PMC4869808 25979090

[B20] Jiang X. Wang G. Lee A. J. Stornetta R. L. Zhu J. J. (2013). The organization of two new cortical interneuronal circuits. *Nat. Neurosci.* 16 210–218. 10.1038/nn.3305 23313910 PMC3589105

[B21] Kawaguchi Y. Kubota Y. (1996). Physiological and morphological identification of somatostatin-or vasoactive intestinal polypeptide-containing cells among GABAergic cell subtypes in rat frontal cortex. *J. Neurosci.* 16 2701–2715. 8786446 10.1523/JNEUROSCI.16-08-02701.1996 PMC6578756

[B22] Kawaguchi Y. Kubota Y. (1997). GABAergic cell subtypes and their synaptic connections in rat frontal cortex. *Cereb. Cortex* 7 476–486. 10.1093/cercor/7.6.476 9276173

[B23] Kerlin A. M. Andermann M. L. Berezovskii V. K. Reid R. C. (2010). Broadly tuned response properties of diverse inhibitory neuron subtypes in mouse visual cortex. *Neuron* 67 858–871. 10.1016/j.neuron.2010.08.002 20826316 PMC3327881

[B24] Kvitsiani D. Ranade S. Hangya B. Taniguchi H. Huang J. Z. Kepecs A. (2013). Distinct behavioural and network correlates of two interneuron types in prefrontal cortex. *Nature* 498 363–366. 10.1038/nature12176 23708967 PMC4349584

[B25] Lee S. Hjerling-Leﬄer J. Zagha E. Fishell G. Rudy B. (2010). The largest group of superficial neocortical GABAergic interneurons expresses ionotropic serotonin receptors. *J. Neurosci.* 30 16796–16808. 10.1523/JNEUROSCI.1869-10.2010 21159951 PMC3025500

[B26] Lee S. Kruglikov I. Huang Z. J. Fishell G. Rudy B. (2013). A disinhibitory circuit mediates motor integration in the somatosensory cortex. *Nat. Neurosci.* 16 1662–1670. 10.1038/nn.3544 24097044 PMC4100076

[B27] Lee S. H. Kwan A. C. Dan Y. (2014). Interneuron subtypes and orientation tuning. *Nature* 508 E1–E2. 10.1038/nature13128 24695313

[B28] Letzkus J. J. Wolff S. B. Meyer E. M. Tovote P. Courtin J. Herry C. (2011). A disinhibitory microcircuit for associative fear learning in the auditory cortex. *Nature* 480 331–335. 10.1038/nature10674 22158104

[B29] Li L. Y. Xiong X. R. Ibrahim L. A. Yuan W. Tao H. W. Zhang L. I. (2014a). Differential receptive field properties of parvalbumin and somatostatin inhibitory neurons in mouse auditory cortex. *Cereb. Cortex* 417 10.1093/cercor/bht417 [Epub ahead of print] PMC4459283 24425250

[B30] Li L. Y. Ji X. Y. Liang F. Li Y. T. Xiao Z. Tao H. W. (2014b). A feedforward inhibitory circuit mediates lateral refinement of sensory representation in upper layer 2/3 of mouse primary auditory cortex. *J. Neurosci.* 34 13670–13683. 10.1523/JNEUROSCI.1516-14.2014 25297094 PMC4188965

[B31] Liu B. H. Li P. Li Y. T. Sun Y. J. Yanagawa Y. Obata K. (2009). Visual receptive field structure of cortical inhibitory neurons revealed by two-photon imaging guided recording. *J. Neurosci.* 29 10520–10532. 10.1523/JNEUROSCI.1915-09.2009 19710305 PMC2779138

[B32] Liu B. H. Li P. Sun Y. J. Li Y. T. Zhang L. I. Tao H. W. (2010). Intervening inhibition underlies simple-cell receptive field structure in visual cortex. *Nat. Neurosci.* 13 89–96. 10.1038/nn.2443 19946318 PMC2818750

[B33] Liu B. H. Li Y. T. Ma W. P. Pan C. J. Zhang L. I. Tao H. W. (2011). Broad inhibition sharpens orientation selectivity by expanding input dynamic range in mouse simple cells. *Neuron* 71 542–554. 10.1016/j.neuron.2011.06.017 21835349 PMC3154747

[B34] Ma W. P. Liu B. H. Li Y. T. Huang Z. J. Zhang L. I. Tao H. W. (2010). Visual representations by cortical somatostatin inhibitory neurons—selective but with weak and delayed responses. *J. Neurosci.* 30 14371–14379. 10.1523/JNEUROSCI.3248-10.2010 20980594 PMC3001391

[B35] Madisen L. Mao T. Koch H. Zhuo J. M. Berenyi A. Fujisawa S. (2012). A toolbox of cre-dependent optogenetic transgenic mice for light-induced activation and silencing. *Nat. Neurosci.* 15 793–802. 10.1038/nn.3078 22446880 PMC3337962

[B36] Markram H. Toledo-Rodriguez M. Wang Y. Gupta A. Silberberg G. Wu C. (2004). Interneurons of the neocortical inhibitory system. *Nat. Rev. Neurosci.* 5 793–807. 10.1038/nrn1519 15378039

[B37] Mata M. L. Ringach D. L. (2005). Spatial overlap of ON and OFF subregions and its relation to response modulation ratio in macaque primary visual cortex. *J. Neurophysiol.* 93 919–928. 10.1152/jn.00668.2004 15371494

[B38] Miyoshi G. Hjerling-Leﬄer J. Karayannis T. Sousa V. H. Butt S. J. Battiste J. (2010). Genetic fate mapping reveals that the caudal ganglionic eminence produces a large and diverse population of superficial cortical interneurons. *J. Neurosci.* 30 1582–1594. 10.1523/JNEUROSCI.4515-09.2010 20130169 PMC2826846

[B39] Moore A. K. Wehr M. (2013). Parvalbumin-expressing inhibitory interneurons in auditory cortex are well-tuned for frequency. *J. Neurosci.* 33 13713–13723. 10.1523/JNEUROSCI.0663-13.2013 23966693 PMC3755717

[B40] Nauhaus I. Nielsen K. J. Callaway E. M. (2012). Nonlinearity of two-photon Ca2+ imaging yields distorted measurements of tuning for V1 neuronal populations. *J. Neurophysiol.* 107 923–936. 10.1152/jn.00725.2011 22114159 PMC3289467

[B41] Niell C. M. Stryker M. P. (2008). Highly selective receptive fields in mouse visual cortex. *J. Neurosci.* 28 7520–7536. 10.1523/JNEUROSCI.0623-08.2008 18650330 PMC3040721

[B42] Pfeffer C. K. Xue M. He M. Huang Z. J. Scanziani M. (2013). Inhibition of inhibition in visual cortex: the logic of connections between molecularly distinct interneurons. *Nat. Neurosci.* 16 1068–1076. 10.1038/nn.3446 23817549 PMC3729586

[B43] Pi H. J. Hangya B. Kvitsiani D. Sanders J. I. Huang Z. J. Kepecs A. (2013). Cortical interneurons that specialize in disinhibitory control. *Nature* 503 521–524. 10.1038/nature12676 24097352 PMC4017628

[B44] Pologruto T. A. Sabatini B. L. Svoboda K. (2003). ScanImage: flexible software for operating laser scanning microscopes. Biomed. Eng. *Online* 2:13 10.1186/1475-925X-2-13 PMC161784 12801419

[B45] Porter J. T. Cauli B. Tsuzuki K. Lambolez B. Rossier J. Audinat E. (1999). Selective excitation of subtypes of neocortical interneurons by nicotinic receptors. *J. Neurosci.* 19 5228–5235. 10377334 10.1523/JNEUROSCI.19-13-05228.1999 PMC6782331

[B46] Rudy B. Fishell G. Lee S. Hjerling-Leﬄer J. (2011). Three groups of interneurons account for nearly 100% of neocortical GABAergic neurons. *Dev. Neurobiol.* 71 45–61. 10.1002/dneu.20853 21154909 PMC3556905

[B47] Runyan C. A. Schummers J. Van Wart A. Kuhlman S. J. Wilson N. R. Huang Z. J. (2010). Response features of parvalbumin-expressing interneurons suggest precise roles for subtypes of inhibition in visual cortex. *Neuron* 67 847–857. 10.1016/j.neuron.2010.08.006 20826315 PMC2948796

[B48] Schumacher J. W. Schneider D. M. Woolley S. M. (2011). Anesthetic state modulates excitability but not spectral tuning or neural discrimination in single auditory midbrain neurons. *J. Neurophysiol.* 106 500–514. 10.1152/jn.01072.2010 21543752 PMC3154814

[B49] Skottun B. C. Grosof D. H. De Valois R. L. (1991). On the responses of simple and complex cells to random dot patterns. *Vision Res.* 31 43–46. 10.1016/0042-6989(91)90071-C 2006552

[B50] Sun Y. J. Kim Y. J. Ibrahim L. A. Tao H. W. Zhang L. I. (2013). Synaptic mechanisms underlying functional dichotomy between intrinsic-bursting and regular-spiking neurons in auditory cortical layer 5. *J. Neurosci.* 33 5326–5339. 10.1523/JNEUROSCI.4810-12.2013 23516297 PMC3714095

[B51] Sun Y. J. Wu G. K. Liu B. H. Li P. Zhou M. Xiao Z. (2010). Fine-tuning of pre-balanced excitation and inhibition during auditory cortical development. *Nature* 465 927–931. 10.1038/nature09079 20559386 PMC2909826

[B52] Sutter M. L. Schreiner C. E. (1991). Physiology and topography of neurons with multipeaked tuning curves in cat primary auditory cortex. *J. Neurophysiol.* 65 1207–1226. 1869913 10.1152/jn.1991.65.5.1207

[B53] Tan A. Y. Atencio C. A. Polley D. B. Merzenich M. M. Schreiner C. E. (2007). Unbalanced synaptic inhibition can create intensity-tuned auditory cortex neurons. *Neuroscience* 146 449–462. 10.1016/j.neuroscience.2007.01.019 17320296

[B54] Taniguchi H. He M. Wu P. Kim S. Paik R. Sugino K. (2011). A resource of Cre driver lines for genetic targeting of GABAergic neurons in cerebral cortex. *Neuron* 71 995–1013. 10.1016/j.neuron.2011.07.026 21943598 PMC3779648

[B55] von Engelhardt J. Eliava M. Meyer A. H. Rozov A. Monyer H. (2007). Functional characterization of intrinsic cholinergic interneurons in the cortex. *J. Neurosci.* 27 5633–5642. 10.1523/JNEUROSCI.4647-06.2007 17522308 PMC6672773

[B56] Wang Y. Gupta A. Toledo-Rodriguez M. Wu C. Z. Markram H. (2002). Anatomical, physiological, molecular and circuit properties of nest basket cells in the developing somatosensory cortex. *Cereb. Cortex* 12 395–410. 10.1093/cercor/12.4.395 11884355

[B57] Wilson N. R. Runyan C. A. Wang F. L. Sur M. (2012). Division and subtraction by distinct cortical inhibitory networks in vivo. *Nature* 488 343–348. 10.1038/nature11347 22878717 PMC3653570

[B58] Wu G. K. Arbuckle R. Liu B. H. Tao H. W. Zhang L. I. (2008). Lateral sharpening of cortical frequency tuning by approximately balanced inhibition. *Neuron* 58 132–143. 10.1016/j.neuron.2008.01.035 18400169 PMC2447869

[B59] Wu G. K. Li P. Tao H. W. Zhang L. I. (2006). Nonmonotonic synaptic excitation and imbalanced inhibition underlying cortical intensity tuning. *Neuron* 52 705–715. 10.1016/j.neuron.2006.10.009 17114053 PMC1764440

[B60] Xiong X. R. Liang F. Li H. Mesik L. Zhang K. K. Polley D. B. (2013). Interaural level difference-dependent gain control and synaptic scaling underlying binaural computation. *Neuron* 79 738–753. 10.1016/j.neuron.2013.06.012 23972599 PMC3755964

[B61] Xu X. Callaway E. M. (2009). Laminar specificity of functional input to distinct types of inhibitory cortical neurons. *J. Neurosci.* 29 70–85. 10.1523/JNEUROSCI.4104-08.2009 19129386 PMC2656387

[B62] Xu X. Roby K. D. Callaway E. M. (2010). Immunochemical characterization of inhibitory mouse cortical neurons: three chemically distinct classes of inhibitory cells. *J. Comp. Neurol.* 518 389–404. 10.1002/cne.22229 19950390 PMC2804902

[B63] Zhang L. I. Zhou Y. Tao H. W. (2011). Perspectives on: information and coding in mammalian sensory physiology: inhibitory synaptic mechanisms underlying functional diversity in auditory cortex. *J. Gen. Physiol.* 138 311–320. 10.1085/jgp.201110650 21875980 PMC3171074

[B64] Zhang S. Xu M. Kamigaki T. Do J. P. H. Chang W. C. Jenvay S. (2014). Long-range and local circuits for top-down modulation of visual cortex processing. *Science* 345 660–665. 10.1126/science.1254126 25104383 PMC5776147

[B65] Zhou M. Tao H. W. Zhang L. I. (2012). Generation of intensity selectivity by differential synaptic tuning: fast-saturating excitation but slow-saturating inhibition. *J. Neurosci*. 32 18068–18078. 10.1523/JNEUROSCI.3647-12.2012 23238722 PMC3685279

